# Pediatric Patients with Sitosterolemia: Next-Generation Sequencing and Biochemical Examination in Clinical Practice

**DOI:** 10.3390/jpm13101492

**Published:** 2023-10-14

**Authors:** Valentina V. Miroshnikova, Petr A. Vasiluev, Svetlana V. Linkova, Vladislav M. Soloviov, Olga N. Ivanova, Ekaterina R. Tolmacheva, Vasilisa Y. Udalova, Polina V. Baranova, Darya Y. Aleksandrova, Tatiana V. Strokova, Irina M. Miklashevich, Artem D. Izumchenko, Kseniia V. Dracheva, Maria N. Grunina, Nataliya N. Smirnova, Anna S. Kuchina, Ekaterina Y. Zakharova, Sofya N. Pchelina

**Affiliations:** 1 Scientific Research Center, Pavlov First Saint-Petersburg State Medical University, Saint-Petersburg 197022, Russia; artemiz98@yandex.ru (A.D.I.); fatal-ks@mail.ru (K.V.D.); nephro-uro-kids@mail.ru (N.N.S.); 2 Petersburg Nuclear Physics Institute Named by B.P. Konstantinov of National Research Centre “Kurchatov Institute”, Gatchina 188300, Russia; by2306@mail.ru; 3 Research Center for Medical Genetics, Moscow 115522, Russia; vasiluev1993@yandex.ru (P.A.V.); baranova.pv@gmail.com (P.V.B.); dar.alex.ur@gmail.com (D.Y.A.); kuchina@med-gen.ru (A.S.K.); doctor.zakharova@gmail.com (E.Y.Z.); 4 Children Municipal Multi-Specialty Clinical Center of High Medical Technology Named after K.A. Rauhfus, Saint-Petersburg 191036, Russia; 5 Veltischev Research and Clinical Institute for Pediatrics and Pediatric Surgery, Pirogov Russian National Research Medical University, Moscow 125412, Russiaimiklashevich@pedklin.ru (I.M.M.); 6 “National Medical Research Center for Obstetrics, Gynecology and Perinatology” of the Ministry of Health of the Russian Federation, Moscow 117198, Russia; tetisae@gmail.com; 7 LLC “Genomed”, Moscow 105005, Russia; 8 Federal Reresearch Centre of Nutrition and Biotechnology, Moscow 109240, Russia; strokova_t.v@mail.ru

**Keywords:** sitosterolemia, ABCG8 variants, phytosterols, NGS

## Abstract

Here, we report the pediatric cases of sitosterolemia, a rare autosomal-recessive genetic disorder, characterized by high concentrations of plant sterols in blood and heterogeneity manifestations. All three patients (two girls aged 2 and 6 years old, and one boy aged 14 years old) were initially diagnosed with hypercholesterinemia. Next-generation sequencing (NGS) revealed homozygous (p.Leu572Pro/p.Leu572Pro) and compound (p.Leu572Pro/p.Gly512Arg and p.Leu572Pro/p.Trp361*) variants in the *ABCG8* gene that allowed for the diagnosis of sitosterolemia. Two patients whose blood phytosterol levels were estimated before the diet demonstrated high levels of sitosterol/campesterol (69.6/29.2 and 28.3/12.4 μmol/L, respectively). Here, we demonstrate that NGS-testing led to the proper diagnosis that is essential for patients’ management. The variant p.Leu572Pro might be prevalent among patients with sitosterolemia in Russia.

## 1. Introduction

Sitosterolemia (OMIM#210,250/618666) is a rare autosomal recessive disorder caused by homozygous or compound heterozygous genetic variants in the *ABCG5* and *ABCG8* genes, encoding the ATP-binding cassette (ABC) subfamily G members 5 and 8 (ABCG5 and ABCG8), respectively. Approximately 200 cases have been reported today worldwide [1]. ABCG5 and ABCG8 proteins form a heterodimer that functions as a transporter of sterols out of the cells to the intestinal lumen or into the bile. The proteins are expressed only in hepatocytes, gallbladder epithelium, and enterocytes and are responsible for extraction of sterols with plant sterol preferred over cholesterol [2]. ABCG5 and ABCG8 deficiencies result in a severe accumulation of plant sterols (phytosterols) in plasma and tissues.

Sitosterolemia is phenotypically heterogeneous, and the disease clinical manifestation could be highly diverse. Some individuals with homozygous mutations may appear almost totally asymptomatic, while others may manifest with variable symptoms including premature death due to cardiovascular complications [3]. The major clinical manifestation of sitosterolemia as discussed today are xanthomas [1]. As some sitosterolemic patients have elevated cholesterol levels, misdiagnosis of sitosterolemia with familial hypercholesterolemia may be rather prevalent and lead to the treatment that could even worthen the disease progression [4,5]. However, patients with sitosterolemia may present with normal cholesterol levels as well how it was described in the first cases [6]. Sitosterolemia can also been presented with hematological abnormalities—anemia and thrombocytopenia. Eventually, years could pass until patients receive the proper diagnosis and treatment [5,7]. Thereafter, NGS with the custom panels including *ABCG5* and *ABCG8* and biochemical phytosterol evaluation are the approaches that allow for precise diagnosis. Proper management of pediatric patients with sitosterolemia will allow for the escaping from complications of the disease such as cardiovascular disease and others in adulthood. Here, we present clinical, biochemical, and genetic descriptions of the three newly identified pediatric patients with sitosterolemia.

## 2. Materials and Methods

Genetic testing was conducted with NGS custom panels according to protocols of the genetic laboratories. For the 3 described clinical cases, different NGS custom panels were used, but they all included genes of monogenic hypercholesterolemia as well as the *ABCG5* and *ABCG8* genes for differential diagnosis of sitosterolemia.

Case 1: Genetic testing was conducted in LCC “Genomed”. The DNA library was enriched using a selective capture method targeting the protein-coding regions of 18 human genes with known clinical significance: *ABCA1, ABCG5, ABCG8, APOA1, APOA5, APOB, APOC2, APOC3, APOE, CETP, GPIHBP1, LDLR, LDLRAP1, LIPC, LPL, PCSK9, SLCO1B1,* and *SREBF2*. Paired-end sequencing was carried out on an Illumina NextSeq 500 with average coverage 117.2× and read length 2 × 151 b.p. The pipeline employs BWA MEM for sequence alignment; Freebayes for variant calling; and SnpEff and SnpSift tools for variant annotation, using the default settings except for Freebayes where long haplotype calling was turned off (--haplotype-length 0 --use-best-n-alleles 2). Raw calls were filtered based on sequencing depth (10 or above to pass filter) and strand bias (required presence of at least one alternative allele read on both DNA strands in calls where reference reads are present on both strands).

Case 2: Genetic testing was conducted in Pavlov First Saint-Petersburg State Medical University. Library was prepared using an Illumina compatible reagent kit Prep&Seq (Parseq Lab Co., Saint-Petersburg, Russia) with the custom panel for differential diagnosis of hereditary dyslipidemias (VariFind LM assay IL-v1.1.1: *ABCA1, ABCG1, ABCG5, ABCG8, ANGPTL3, APOA1, APOA4, APOA5, APOB, APOC2, APOC3, APOE, CETP, CREB3L3, CYP27A1, CYP7A1, GCK, GPD1, GPIHBP1, HNF1A, LCAT, LDLR, LDLRAP1, LIPA, LIPC, LIPG, LMF1, LPL, LRP6, MTTP, MYLIP, NPC1L1, PCSK9, PNPLA5, SAR1B, SCARB1, SORT1, STAP1,* and *TTR*; Parseq Lab Co., Saint-Petersburg, Russia). Paired-end sequencing (2 × 150 b.p.) was performed on a MiSeq instrument (Illumina, San Diego, CA USA). Fastq files were analyzed by the manufacturer’s VariFind Software v2.2 (Parseq Lab Co., Russia). Variant validation was performed by PCR-direct sequencing using the ABI BigDye Terminator 3.1 kit on a Nanofor-05 instrument (Syntol, Moscow, Russia).

Case 3: Genetic testing was conducted in Research Center for Medical Genetics. Isolation of genomic DNA was carried out from whole blood using the DNAEasy (QiaGen, Venlo, the Netherlands), according to the manufacturer’s standard protocol. DNA libraries were constructed with the AmpliSeq custom panel (Thermo Fisher Scientific, Inc., Waltham, MA, USA; cat. Nos. 04779971_Dyslipidemia_IAD175748_182: *ABCA1, ABCG1, ABCG5, ABCG8, AGPAT2, ALMS1, ANGPTL3, APOA1, APOA2, APOA4, APOA5, APOB, APOC1, APOC2, APOC3, APOE, APOH, BSCL2, CAV1, CAV2, CAVIN1, CETP, CH25H, CIDEC, COQ2, CPT2, CREB3L3, GCK, GPD1, GPIHBP1, HNF1A, LCAT, LDLR, LDLRAP1, LIPA, LIPC, LIPE, LIPG, LMF1, LMNA, LMNB2, LPA, LPL, MTTP, MYLIP, NPC1, NPC1L1, NPC2, PCSK9, PLIN1, PLTP, PP1R17, PPARA, PPARG, PYGM, SAR1B, SCARB1, SLCO1B1, SLCO1B3,* and *STAP1)*. Paired-end sequencing (2 × 300 b.p.) was carried out on an Illumina MiSeq instrument (MiSeq Reagent Kit v3 (600 cycle)). To annotate the identified variants, nomenclature presented on the site http://varnomen.hgvs.org/recommendations/DNA version 20.05 accessed on 1 July 2023 was used. Validation of the identified variants in proband and genotyping of parents were carried out by automated Sanger sequencing according to the manufacturer’s protocol on the ABIPrism 3500 xl device (Applied Biosystems, Foster City, CA, USA).

For phytosterol blood level evaluation, we applied gas chromatography–mass spectrometry (GC-MS) TQ-8050 (Shimadzu, Nakagyo-ku, Japan) with autosampler AOC-20i, HP-5MS. Sample preparation and analysis conditions were performed with GC-MS according to Joon Hee Lee et al. [8].

## 3. Results

### 3.1. Case 1

A one-year-old girl was initially examined by a dermatologist in 2016. The reason was dense formations of yellow-orange color—which were assessed by the doctor as xanthomas—on the knee joints, elbow joints, and gluteal folds. Hypercholesterolemia was revealed with a maximum level of total cholesterol of 16.0 mmol/L. Echocardiography was without structural and hemodynamic changes. The abdominal ultrasound showed no hepatosplenomegaly or fatty liver. Thrombocytopenia was not observed. Extracranial color triplex scanning brachiocephalic arteries was not performed. Homozygous familial hypercholesterolemia was suspected. However, both parents had no dyslipidemia or signs of premature coronary artery disease (CAD). Plasmapheresis was conducted without marked efficiency. Targeted sequencing with a custom panel, which additionally included genes of sitosterolemia *ABCG5* and *ABCG8*, revealed that the patient was homozygous for a missense variant in the *ABCG8* gene rs769576789 NM_022437.3:c.1715T>C p.(Leu572Pro) (Table 1). These variants were confirmed by Sanger sequencing, and parents were heterozygous. So, a diagnosis of sitosterolemia was applied, and diet recommendations were prescribed. Phytosterol levels in blood were determined only after diet at the age of 8 (Table 1). After long dietary restriction, sitosterol and campesterol levels were 13.8 μmol/L and 7.0 μmol/L, respectively.

### 3.2. Case 2

A six-year-old girl initially was examined by an endocrinologist. Hormone levels were normal. Hypercholesterolemia was detected with the maximal total cholesterol level of 12.6 mmol/L. The reason was premature pubarche and strong odor of sweat from the armpits from the age of four as a possible sign of premature adrenarche. Targeted sequencing revealed two rare variants in the *ABCG8* gene: rs769576789 NM_022437.3:c.1715T>C p.(Leu572Pro) and rs376069170 NM_022437.3:c.1534G>A p.(Gly512Arg), which both were confirmed by Sanger sequencing (Table 1; Supplementary File S1). The proband’s mother and sibling had only one variant—p.(Gly512Arg). There are no data on pathogenicity of this variant in public databases. As the proband was compound heterozygous for these two variants, this was reason to suspect sitosterolemia. A multiplex sitosterol assay demonstrated that plant sterol levels in the proband’s serum were markedly elevated: sitosterol 28.3 μmol/L; campesterol 12.4 μmol/L. Any other signs characteristic of patients with sitosterolemia were not observed. Xanthoma was not found on the skin. Extracranial color triplex scanning of the brachiocephalic arteries showed that the intima-media complex was not thickened, there were no signs of angiodystonia, and no hemodynamically significant disorders detected; the peculiarity of the course was that in the left vertebral artery and the high entry of the right vertebral artery into the bone canal, the venous outflow was not disturbed. Echocardiography was without obvious structural and hemodynamic changes. The abdominal ultrasound showed no hepatosplenomegaly or fatty liver. Thrombocytopenia was not observed. Among concomitant diagnosis, angiopathy of the retinal vessels and atopic dermatitis were noted. NGS testing allowed the diagnosis to be made within half a year after contacting a doctor. The initial diet resulted in total cholesterol level of 5.5 mmol/L.

### 3.3. Case 3

A 14-year-old boy had a history of elevated total cholesterol levels from the age of 4. First, the patient was referred to the clinic with knee xanthomas. The total cholesterol level was 12 mmol/L, which led to the diagnosis of hypercholesterolemia. Knee xanthomas were resected. A cholesterol restriction diet resulted in total cholesterol decrease to 7 mmol/L. At his extended examination at the age of 14 years old, the proband had clinical signs of thrombocytopenia and xanthomatosis. The patient also demonstrated hepatosplenomegaly, as well as several cardiac complications (aortic heart valve insufficiency of the first degree, the formation of supravalvular stenosis, mitral insufficiency of the second degree). Ultrasound and echocardiography examination did not show any signs of atherosclerosis. Targeted sequencing revealed two rare variants in the *ABCG8* gene: rs769576789 NM_022437.3:c.1715T>C p.(Leu572Pro) and rs137852987 NM_022437.3: c.1083G>A p.(Trp361*) (Table 1; Supplementary File S1). These variants were confirmed by Sanger sequencing, and parents were heterozygous. Both variants are registered in HGMD (2022.1) as pathogenic in patients with sitosterolemia (CM012318 and CM003582, respectively). The mother and siblings were found to have normal cholesterol, and the father was found to have a transient increase corrected by diet. A multiplex sitosterol assay demonstrated that plant sterol levels in the proband’s serum were markedly elevated: sitosterol 69.6 μmol/L; campesterol 29.2 μmol/L.

## 4. Discussion

Here, we present three new cases of sitosterolemia where gene sequencing and plasma plant sterol measurement allowed for the correct diagnosis in pediatric patients. The three case reports describe sitosterolemia in an 8-year-old girl, a 6-year-old girl, and a 14-year-old boy with first disease manifestation in 1, 6, and 4 years, respectively (case 1, case 2, and case 3, respectively). The disease presentation was different. In cases 1 and 3, sitosterolemia manifested with xanthomas and increased levels of total and LDL cholesterol, as opposed to in case 2, with high levels of cholesterol only. The location of the xanthomas in cases 1 and 3 was characteristic: knee joints, elbow joints, and gluteal folds. Xanthomas are the major clinical manifestations of sitosterolemia including pediatric patients [9], and frequently they are the course of an application for medical care. However, it should be noted that xanthomas could be absent. This was the case in case 2: the primary reason for visiting the clinic was premature pubarche, and later hypercholesterolemia was discovered, however, with no xanthomas on the skin. Thus, in the present study, all three pediatric patients exhibited a high cholesterol level, and the diagnosis of familial hypercholesterinemia was initially suspected. This is in agreement with recent research demonstrating that the misdiagnosis of sitosterolemia with familial hypercholesterolemia may be prevalent [10,11].

In all our three cases, the diagnosis was made as a result of molecular-genetic testing that was conducted with NGS custom panels (a different panel in each case). It is important to indicate that these panels included the *ABCG5* and *ABCG8* genes that led to the diagnosis of sitosterolemia that later was supported with a biochemical examination on blood phytosterols levels.

As sitosterolemia is usually misdiagnosed as hypercholesterolemia or hematopathy, or other disorders in some cases, the delay between symptom onset and proper diagnosis could be more than 20 years [7,12]. Phenotypic diversity and concomitant disease make diagnosis difficult. Cases of sitosterolemia with sinus arrhythmia and adrenal insufficiency have been reported [13,14]. The delayed diagnosis is the missed opportunity for a patient to receive prompt therapy, resulting in a number of life-threatening complications, such as premature atherosclerosis and liver function damage caused by drugs and even splenectomy [15].

It is worth noting that genetic testing in our case 1 and case 2 allowed for the establishment of the diagnosis of sitosterolemia during the first year of symptom onset. One should keep in mind that NGS panels for genetic testing of a dyslipidemic condition should include the *ABCG5* and *ABCG8* genes. In that respect, the case of seventeen years of misdiagnosis of sitosterolemia in a 20-year-old woman is indicative, as in the age of 10, the familial dyslipidemia was suspected, but the conducted genetic testing included only all mutations in the LDL receptor and apolipoprotein genes. Eventually, it takes 10 years more to make the diagnosis of sitosterolemia after new extended testing with another NGS panel [4].

Emphasizing the importance of NGS testing, it is worth noting that in the case of rare non-synonymous substitutions in the coding region that was not previously described in sitosterolemia patients and could be referred to only as a variant of unknown significance, the diagnosis could be made only after biochemical examinations of blood phytosterols. The main phytosterols are sitosterol, campesterol, and stigmasterol, with sitosterol being the most prevalent in the diet. Plant sterols are structurally similar to cholesterol, making it difficult to distinguish between cholesterol and phytosterols with routine colorimetric methods. In the present study, gas chromatography–mass spectrometry (GC-MS) was applied for the determination of plasma plant sterols (sitosterol, campesterol, and stigmasterol). As expected, the main differences compared to controls were detected for sitosterol concentrations. According to Tada et al., serum sitosterol ≥ 1 mg/dL (10 µg/mL) is compatible with the diagnosis sitosterolemia [16]. In our study, in cases 2 and 3, their blood phytosterol levels were estimated before the diet and demonstrated levels of sitosterol as 69.6 and 28.3 μmol/L and campesterol as 29.2 and 12.4 μmol/L, respectively. Nonetheless, plasma sitosterol concentrations in homozygous or compound heterozygous forms were overall 10–25 times higher in individuals with sitosterolemia than in healthy controls. However, the influence on the type of mutations on the sitosterol plasma level could not be excluded. We can assume that nonsense variants are associated with higher phytosterol levels, as was found in case 2, where the proband had the p.(Trp361*) variant and a sitosterol concentration of 69.6 μmol/L. The sitosterol concentration was as high as 54.4 µmol/L in adult patients homozygous for p.(Trp361*), as was described earlier [5].

It could be assumed that clinical heterogeneity of sitosterolemia could be explained both with a large variety of plant sterols present in the human diet [17] and a type of genetic variant detected. About 80 variants are reported today in the *ABCG5* and *ABCG8* genes, with 38 in *ABCG5* and 42 in *ABCG8,* with *ABCG8* variants more common among Europeans [1,13]. Nonsense and missense variants are the most frequent. In our study, all probands had *ABCG8* variants, and it is worth noting that they all had the p.(Leu572Pro) variant. A homozygous patient with sitosterolemia aged 30 years old with p.(Leu572Pro) was described earlier [18]. The main sign was elevated LDL cholesterol, so the patient was diagnosed with dyslipidemia, but statins were not effective. He never required any investigations for cardiovascular diseases. On examination at the clinic, a small xanthoma was noted at the elbow tendon, but no xanthelasma or organomegaly. The patient’s platelet count and platelet volume varied between normal and slightly abnormal. Approximately 3 months after starting ezetimibe treatment, there was a marked improvement in his LDL cholesterol. This amino acid substitution as another pathogenic variant p.(Gly574Arg) is located near the apice of transmembrane helix 5 of the protein and involved in contacts with the extracellular domain. This conformational change was supposed to be important for sterol exit from the transmembrane domain (TMD) [19,20]. p.(Gly512Arg) was not earlier linked to sitosterolemia in the ClinVar database. It is very rare substitution with the frequency of 0.000029 according to GnomAD. Still, this variant was found in patients with familial hypercholesterolemia negative for mutations in the canonical *LDLR, APOB,* and *PCSK9* genes [21]. The compound c.323-1G>C/p.(Gly512Arg) was described in a 3-year-old Chinese girl with xanthomas and a sitosterol level of 9.9 μmol/L [22]. However, amino acid substitution in 512 is located in the transmembrane α-helix domain, and hydrophilic uncharged glycine changes to charged arginine, with this being able to destabilize the hydrophobic core of TMD. Other variants in TMD were also described [23]. The most pathogenic variants in *ABCG5* or *ABCG8* are believed to influence the formation of ABCG5/ABCG8 heterodimers and thus prevent effective sterol transport. 

It is interesting to note that increased risk of premature CAD in heterozygous carriers of mutation in the *ABCG5* gene was reported [24], and the frequency of *ABCG5/ABCG8* heterozygous carriers was 8.3 times higher among patients with familial hypercholesterolemia compared to controls [25]. We previously also reported heterozygous adult patients with nonsense variants in *ABCG5* rs199689137 NM_022436.3: c.1336C>T p.(Arg446*) and *ABCG8* rs137852987 NM_022437.3: c.1083G>A p.(Trp361*), with familial hypercholesterolemia and cardiovascular complications [26]. Overall, increased plant sterol and LDL levels in heterozygous carriers of *ABCG5/ABCG8* pathogenic variants could be suspected.

The management of sitosterolemia is aimed at reducing the concentration of plant sterols and cholesterol levels. The correct diagnosis allowed for the escape from years of improper treatment. In addition to plant sterol dietary restriction, the pharmacotherapy with ezetimibe can lead to the reduction of blood phytosterol concentrations [1]. It is interesting to note that unlike familial hypercholesterinemia in sitosterolemia, xanthomas could disappear [27,28].

## 5. Conclusions

In conclusion, our study allowed for the highlighting of several important items. First, the wide variability of clinical manifestations and the similarities with familial hypercholesterolemia contribute to the underestimated prevalence of sitosterolemia. For differential diagnostics, genetic testing should be conducted with NGS panels with *ABCG5* and *ABCG8*, with subsequent testing for blood phytosterol level if such possibility exists. Since specific laboratory methods are required to evaluate plant sterol levels, NGS diagnostics may be at present helpful in timely diagnostics. However, a multiplex sitosterol assay allowed not only for the diagnosis to be made but also for the genotype–phenotype correlations to be found, as well as the monitoring of the results of diet and pharmacotherapy of sitosterolemia.

## Figures and Tables

**Table 1:** Clinical and biochemical characteristics of patients with sitosterolemia including the genetic variants identified by NGS.

Parameter	Case 1	Case 2	Case 3
Current age, years	8	6	14
Sex	female	female	male
Age of first symptoms, years	1	6	4
Total cholesterol range, ref. 2.8–5.1 mmol/L	11.0–16.0	8.5–12.6	7.0–12.0
LDL cholesterol, ref. 1.61–3.61 mmol/L	9.6	9.6	9.0
HDL cholesterol, ref. 0.9–2.0 mmol/L	0.8	1.7	1.3
Triglycerides, ref. 0.31–0.41 mmol/L	1.98	1.4	1.8
Sitosterol, ref. 0.4–3.4 μmol/L	13.8 ^1^	28.3	69.6
Campesterol, ref. 0.1–3.1 μmol/L	7.0 ^1^	12.4	29.2
CVD signs	-	-	-
Xanthomas	+	-	+
Thrombocytopenia	-	-	+
Hepatosplenomegaly	-	-	+
Genetic variant 1	*ABCG8* NM_022437.3: c.1715T>C (p.Leu572Pro) ^2^	*ABCG8* NM_022437.3: c.1715T>C (p.Leu572Pro) ^2^	*ABCG8* NM_022437.3: c.1715T>C (p.Leu572Pro) ^2^
Genetic variant 2	*ABCG8* NM_022437.3: c.1715T>C (p.Leu572Pro) ^2^	*ABCG8* NM_022437.3: c.1534G>A p.(Gly512Arg) ^2^	*ABCG8* NM_022437.3: c.1083G>A (p.Trp361) ^3^

^1^ Possibility to carry out phytosterol concentration analysis appeared only after long-term diet. ^2^ According to the ACMG criteria, the variant is likely pathogenic (PM2 PM1 PP3 PP4). ^3^ According to the ACMG criteria, the variant is pathogenic (PVS1 PM2 PP5 PP4).

## Data Availability

Not applicable.

## Supplementary Materials

The following supporting information can be downloaded at https://www.mdpi.com/article/10.3390/jpm13101492/s1, File S1. Sequencing results.

## References

[1] Rocha V.Z., Tada M.T., Chacra A.P.M., Miname M.H., Mizuta M.H. (2023). Update on Sitosterolemia and Atherosclerosis. Curr. Atheroscler. Rep..

[2] Patel S.B., Graf G.A., Temel R.E. (2018). ABCG5 and ABCG8: More than a defense against xenosterols. J. Lipid Res..

[3] Yoo E.G. (2016). Sitosterolemia: A review and update of pathophysiology, clinical spectrum, diagnosis, and management. Ann. Pediatr. Endocrinol. Metab..

[4] Frederiksen T.C., Mortensen M.B., Kanstrup H.L. (2021). Seventeen years of misdiagnosis in rare dyslipidaemia: A case report of sitosterolaemia in a young female. Eur. Heart J. Case Rep..

[5] Limonova A.S., Ershova A.I., Meshkov A.N., Kiseleva A.V., Divashuk M.G., Kurkina M.V., Drapkina O.M. (2022). Case Report: Next Generation Sequencing in Clinical Practice—A Real Tool for Ending the Protracted Diagnostic Odyssey. Front. Cardiovasc. Med..

[6] Bhattacharyya A.K., Connor W.E. (1974). Beta-sitosterolemia and xanthomatosis. A newly described lipid storage disease in two sisters. J. Clin. Investig..

[7] Wang Z., Cao L., Su Y., Wang G., Wang R., Yu Z., Bai X., Ruan C. (2014). Specific macrothrombocytopenia/hemolytic anemia associated with sitosterolemia. Am. J. Hematol..

[8] Lee J.H., Lee K., Jun S.H., Song S.H., Shin C.H., Song J. (2019). A Multiplex Phytosterol Assay Utilizing Gas Chromatography-Mass Spectrometry for Diagnosis of Inherited Lipid Storage Disorders. Ann. Lab. Med..

[9] Xu L., Wen W., Yang Y., Xie J., Li R., Wu Y., Hu Y., Wang L., Chong M. (2020). Features of Sitosterolemia in Children. Am. J. Cardiol..

[10] Tada H., Okada H., Nomura A., Yashiro S., Nohara A., Ishigaki Y., Takamura M., Kawashiri M.A. (2019). Rare and Deleterious Mutations in ABCG5/ABCG8 Genes Contribute to Mimicking and Worsening of Familial Hypercholesterolemia Phenotype. Circ. J..

[11] Lee J.H., Song D.Y., Jun S.H., Song S.H., Shin C.H., Ki C.S., Lee K., Song J. (2020). High prevalence of increased sitosterol levels in hypercholesterolemic children suggest underestimation of sitosterolemia incidence. PLoS ONE.

[12] Jiang W., Xu Y., Fu Z., Hu M., Wu Q., Ji Y., Li J.Z., Gong Y., Zhou H. (2023). Genetic analysis and functional study of a novel ABCG5 mutation in sitosterolemia with hematologic disease. Gene.

[13] Su S.Q., Xiong D.S., Ding X.M., Kuang J.A., Lin Y.C. (2022). Pediatric patients with familially inherited sitosterolemia: Two case reports. Front. Cardiovasc. Med..

[14] Mushtaq T., Wales J.K., Wright N.P. (2007). Adrenal insufficiency in phytosterolaemia. Eur. J. Endocrinol..

[15] Xia Y., Duan Y., Zheng W., Liang L., Zhang H., Luo X., Gu X., Sun Y., Xiao B., Qiu W. (2022). Clinical, genetic profile and therapy evaluation of 55 children and 5 adults with sitosterolemia. J. Clin. Lipidol..

[16] Tada H., Nomura A., Ogura M., Ikewaki K., Ishigaki Y., Inagaki K., Tsukamoto K., Dobashi K., Nakamura K., Hori M. (2021). Diagnosis and Management of Sitosterolemia 2021. J. Atheroscler. Thromb..

[17] Weingärtner O., Teupser D., Patel S.B. (2015). The Atherogenicity of Plant Sterols: The Evidence from Genetics to Clinical Trials. J. AOAC Int..

[18] Mahzari M.M. (2023). Sitosterolemia: A Case Report and a Concise Literature Review. Case Rep. Endocrinol..

[19] Lee J.Y., Kinch L.N., Borek D.M., Wang J., Wang J., Urbatsch I.L., Xie X.S., Grishin N.V., Cohen J.C., Otwinowski Z. (2016). Crystal structure of the human sterol transporter ABCG5/ABCG8. Nature..

[20] Horenstein R.B., Mitchell B.D., Post W.S., Lütjohann D., von Bergmann K., Ryan K.A., Terrin M., Shuldiner A.R., Steinle N.I. (2013). The ABCG8 G574R variant, serum plant sterol levels, and cardiovascular disease risk in the Old Order Amish. Arterioscler. Thromb. Vasc. Biol..

[21] Lamiquiz-Moneo I., Baila-Rueda L., Bea A.M., Mateo-Gallego R., Pérez-Calahorra S., Marco-Benedí V., Martín-Navarro A., Ros E., Cofán M., Rodríguez-Rey J.C. (2017). ABCG5/G8 gene is associated with hypercholesterolemias without mutation in candidate genes and noncholesterol sterols. J. Clin. Lipidol..

[22] Zhou Z., Su X., Cai Y., Ting T.H., Zhang W., Lin Y., Xu A., Mao X., Zeng C., Liu L. (2022). Features of chinese patients with sitosterolemia. Lipids Health Dis..

[23] Lu K., Lee M.H., Hazard S., Brooks-Wilson A., Hidaka H., Kojima H., Ose L., Stalenhoef A.F., Mietinnen T., Bjorkhem I. (2001). Two genes that map to the STSL locus cause sitosterolemia: Genomic structure and spectrum of mutations involving sterolin-1 and sterolin-2, encoded by ABCG5 and ABCG8, respectively. Am. J. Hum. Genet..

[24] Nomura A., Emdin C.A., Won H.H., Peloso G.M., Natarajan P., Ardissino D., Danesh J., Schunkert H., Correa A., Bown M.J. (2020). Heterozygous *ABCG5* Gene Deficiency and Risk of Coronary Artery Disease. Circ. Genom. Precis. Med..

[25] Tada M.T., Rocha V.Z., Lima I.R., Oliveira T.G.M., Chacra A.P., Miname M.H., Nunes V.S., Nakandakare E.R., Costa Gurgel Castelo M.H., Jannes C.E. (2022). Screening of *ABCG5* and *ABCG8* Genes for Sitosterolemia in a Familial Hypercholesterolemia Cascade Screening Program. Circ. Genom. Precis. Med..

[26] Miroshnikova V.V., Romanova O.V., Ivanova O.N., Fedyakov M.A., Panteleeva A.A., Barbitoff Y.A., Muzalevskaya M.V., Urazgildeeva S.A., Gurevich V.S., Urazov S.P. (2021). Identification of novel variants in the *LDLR* gene in Russian patients with familial hypercholesterolemia using targeted sequencing. Biomed. Rep..

[27] Cheng W.F., Yuen Y.P., Chow C.B., Au K.M., Chan Y.W., Tam S.C. (2003). Sitosterolaemia and xanthomatosis in a child. Hong Kong Med. J..

[28] Niu D.M., Chong K.W., Hsu J.H., Wu T.J., Yu H.C., Huang C.H., Lo M.Y., Kwok C.F., Kratz L.E., Ho L.T. (2010). Clinical observations, molecular genetic analysis, and treatment of sitosterolemia in infants and children. J. Inherit. Metab. Dis..

