# Supplementary material for: Pediatric Patients with Sitosterolemia: Next-Generation Sequencing and Biochemical Examination in Clinical Practice
Source: J Pers Med. 2023 Oct 14;13(10):1492. doi: 10.3390/jpm13101492 (PMC10608675; doi:10.3390/jpm13101492)
Supplement: Supplementary file 1 [file jpm-13-01492-s001.zip › jpm-2615057-supplementary.pdf]

Case 2

Variant 1

chr2:44102330 (GRCh37) NM_022437.3:c.1534G>A p.(Gly512Arg) (rs376069170)

NGS sequencing:

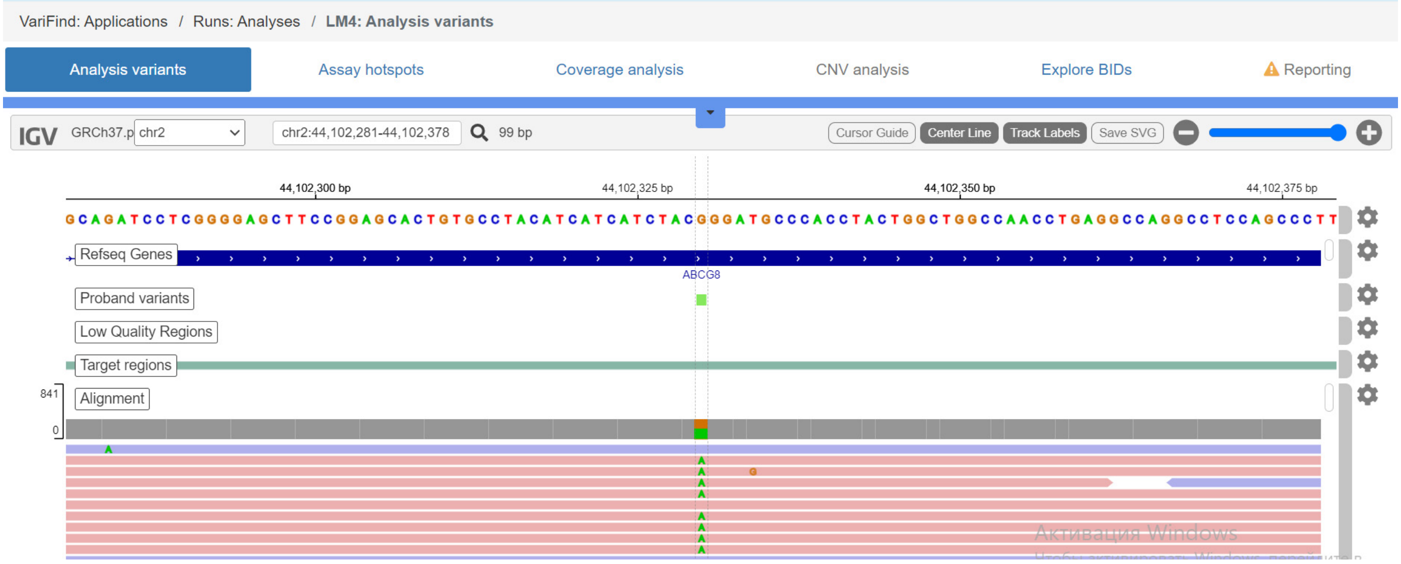

Sanger sequencing results (reverse reads):

Probe identification

Proband - 1

Proband's mother - 2

Proband's sister - 11C

Proband's father DNA – not available

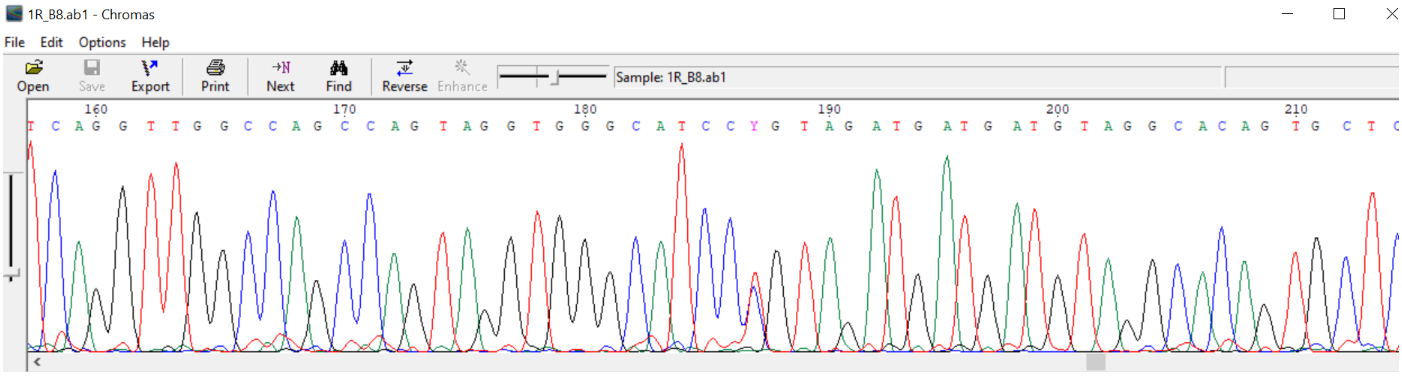

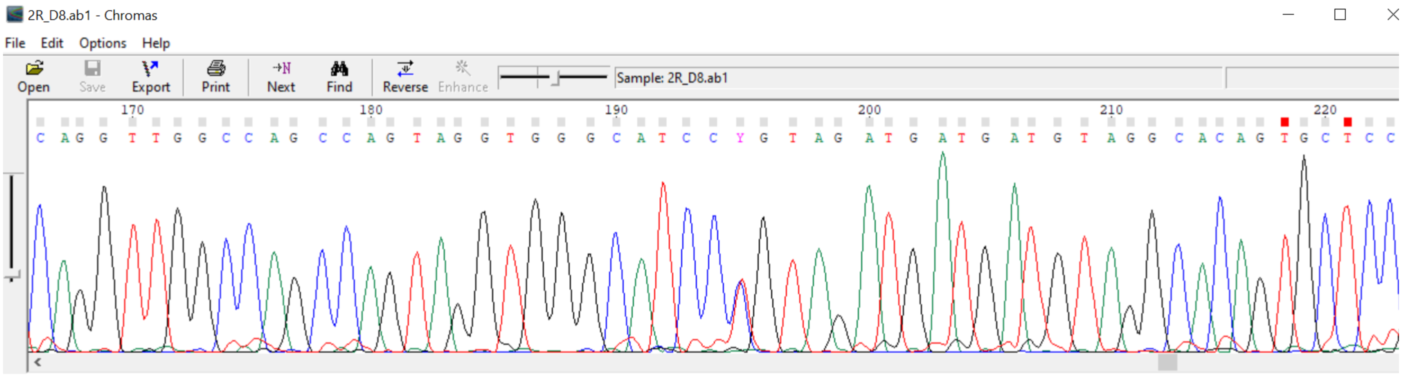

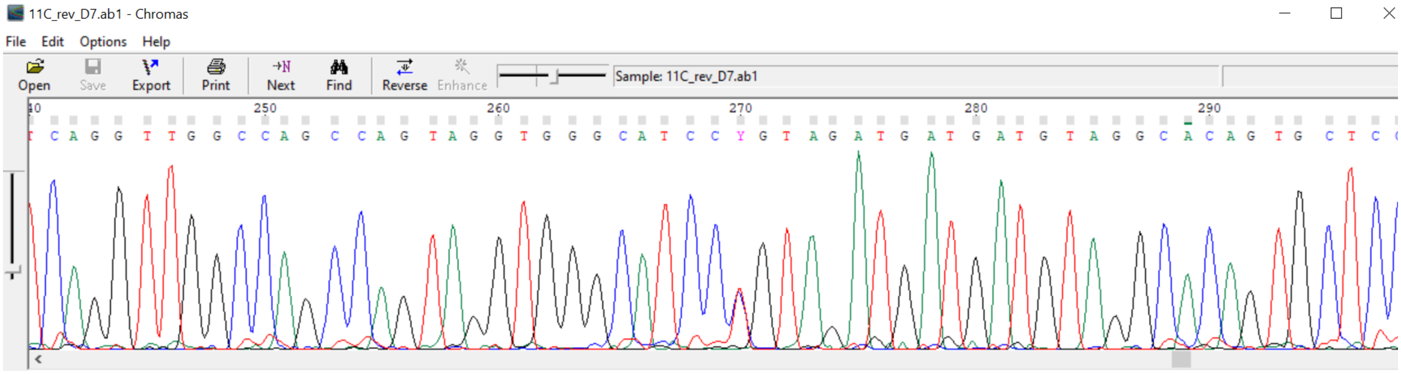

Variant 2

chr2:44102511 (GRCh37) NM_022437.3(ABCG8):c.1715T>C p.(Leu572Pro) (rs769576789)

NGS sequencing:

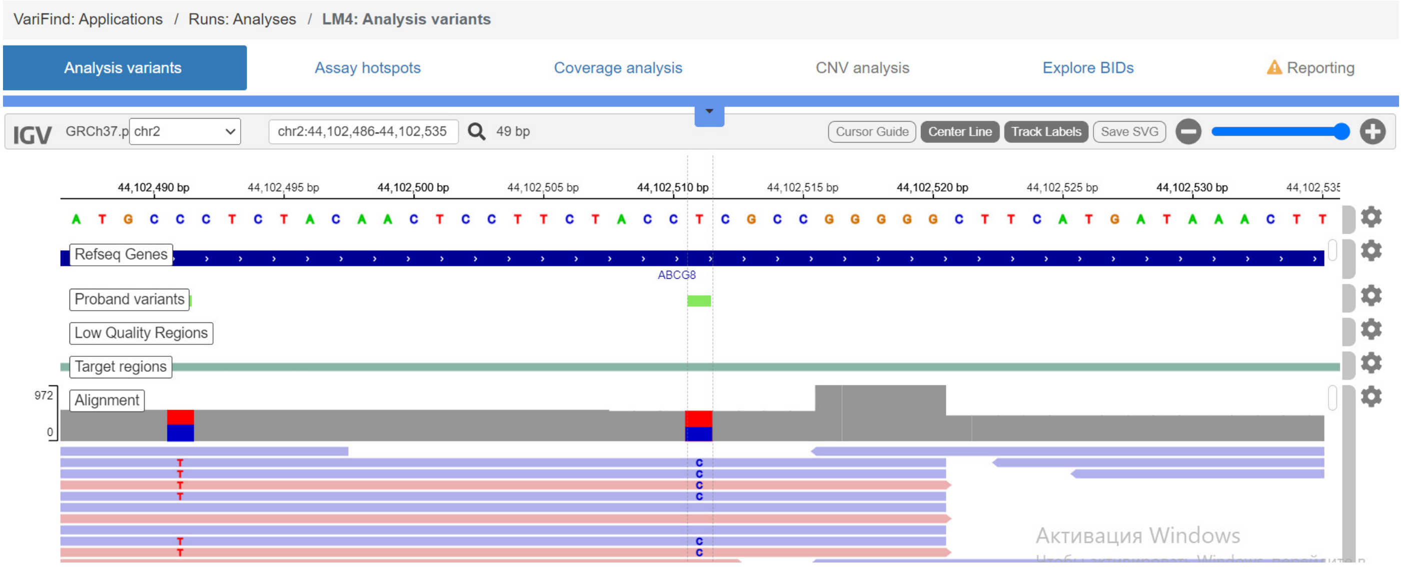

Sanger sequencing results (forward reads):

Probe identification

Proband - 1

Proband’s mother - 2

Proband’s sister - 11C

Proband’s father DNA – not available

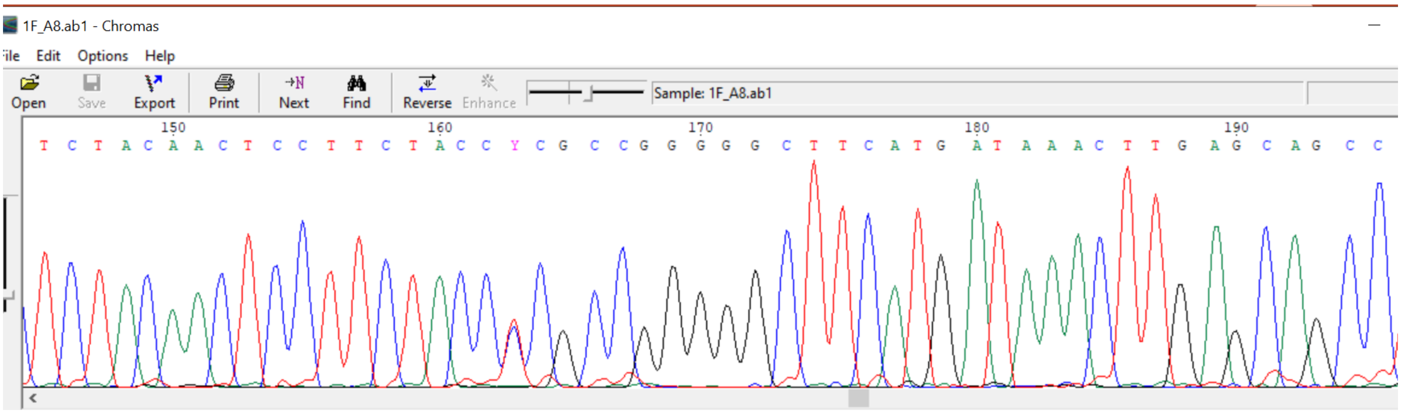

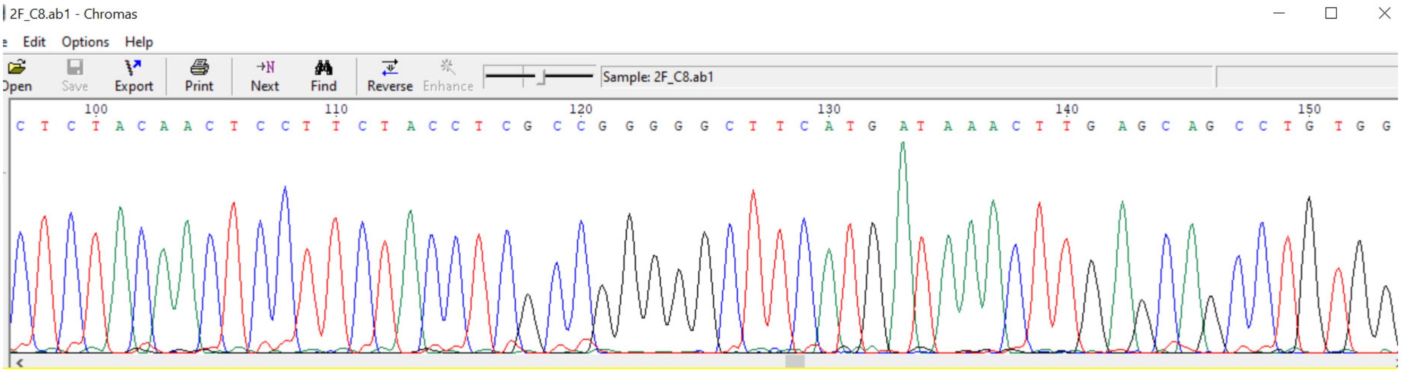

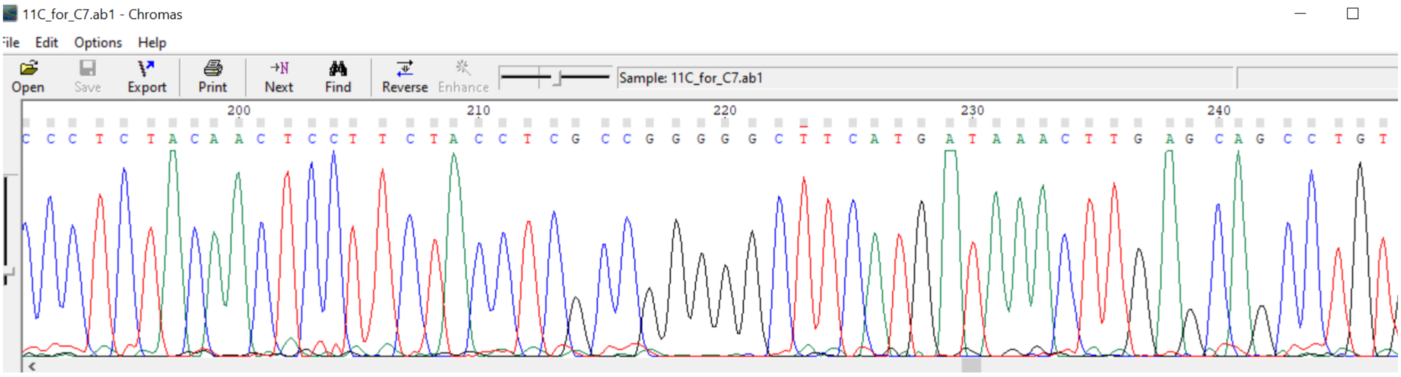

Case 3

ABCG8 chr2:44099233G>A NM_022437.3:c.1083G>A p.Trp361* (rs137852987)

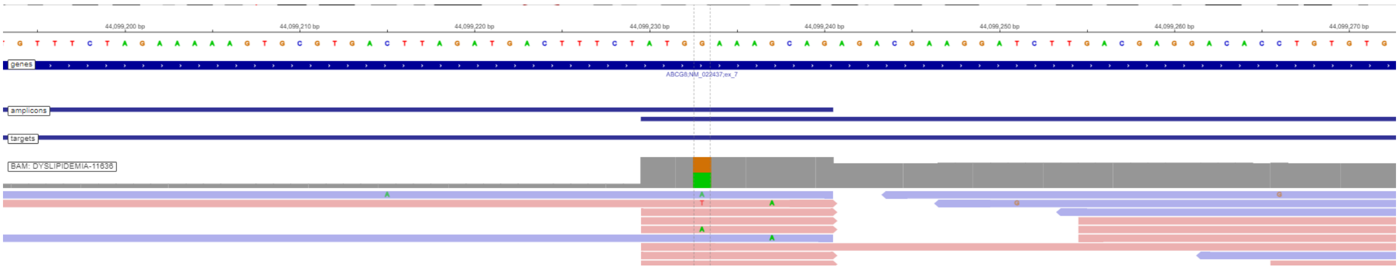

Sanger sequencing results (forward reads):
Probe identification
Proband's mother - DEC7
Proband's father - DAL7
Proband - DAA7

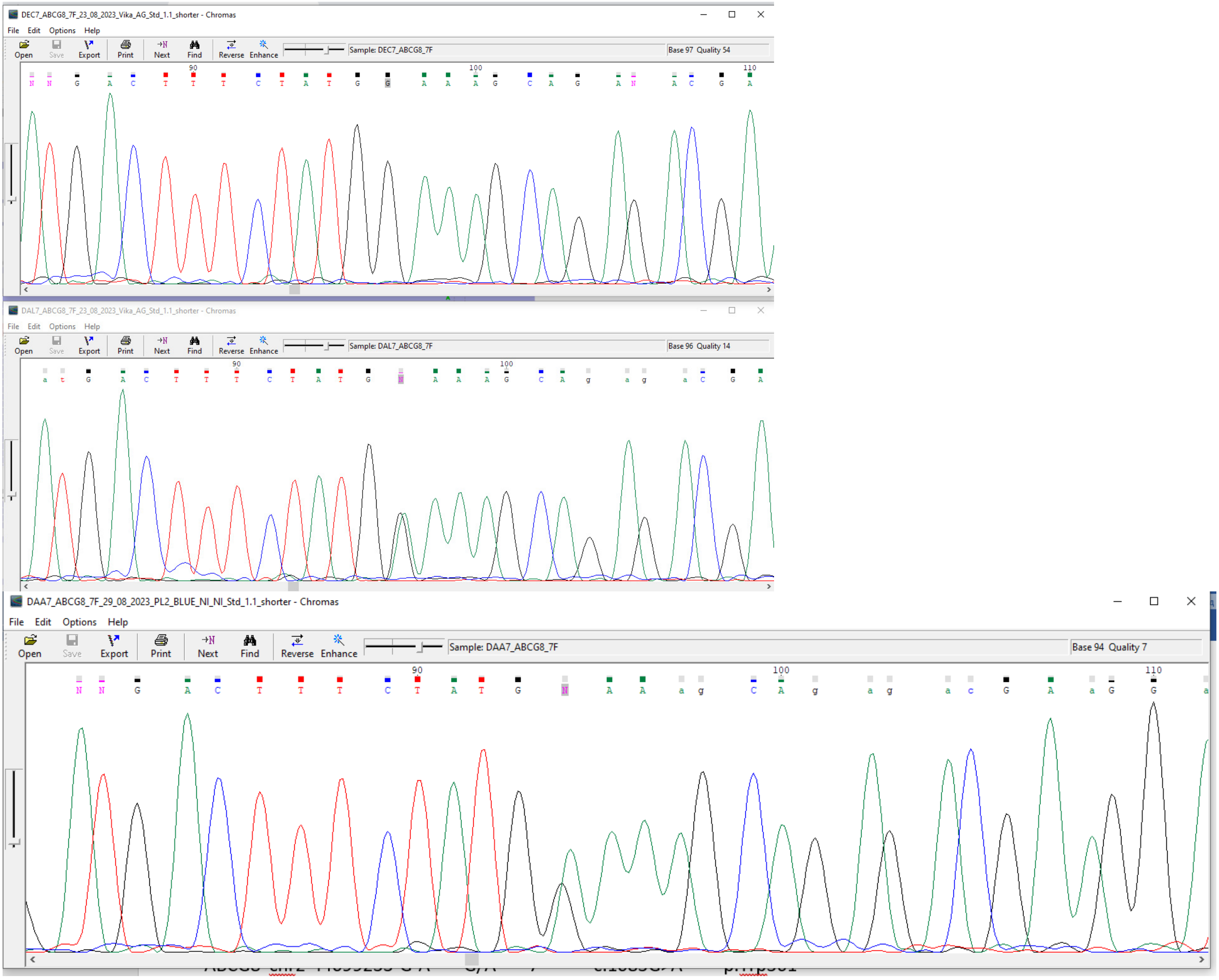

chr2:44102511 (GRCh37) NM_022437.3(ABCG8):c.1715T>C (p.Leu572Pro) (rs769576789)

Sanger sequencing results:
Probe identification
Proband's mother - DEC11
Proband's father - DAL11
Proband - DAA11

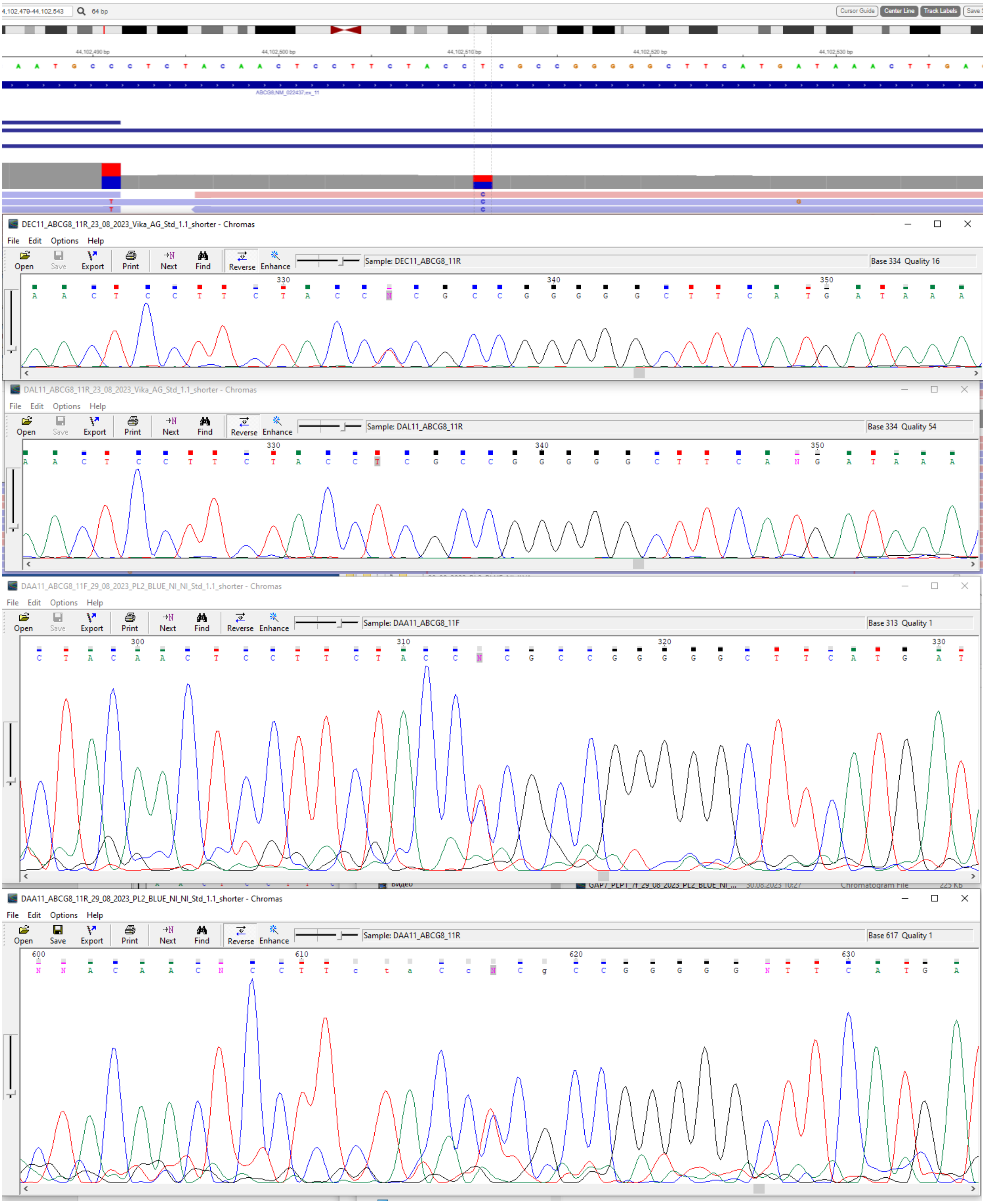

4,102,479-44,102,543
64 bp
Cursor Guide
Center Line
Track Labels
44,102,490 bp
44,102,500 bp
44,102,510 bp
44,102,520 bp
44,102,530 bp
A A T G C C C T C T A C A A C T C C T T C T A C C T C G C C C G G G G G C T T C A T G A T A A A C T T G A
DEC11_ABCG8_11R_23_08_2023_Vika_AG_Std_1.1_shorter - Chromas
File Edit Options Help
Open Save Export Print Next Find Reverse Enhance
Sample: DEC11_ABCG8_11R
Base 334 Quality 16
DAL11_ABCG8_11R_23_08_2023_Vika_AG_Std_1.1_shorter - Chromas
Sample: DAL11_ABCG8_11R
Base 334 Quality 54
DAA11_ABCG8_11F_29_08_2023_PL2_BLUE_NI_NI_Std_1.1_shorter - Chromas
Sample: DAA11_ABCG8_11F
Base 313 Quality 1
DAA11_ABCG8_11R_29_08_2023_PL2_BLUE_NI_NI_Std_1.1_shorter - Chromas
Sample: DAA11_ABCG8_11R
Base 617 Quality 1
